# The Multifactorial Burden of Endometriosis: Predictors of Quality of Life

**DOI:** 10.3390/jcm14020323

**Published:** 2025-01-07

**Authors:** Tomas Kupec, Lieven Nils Kennes, Rebecca Senger, Philipp Meyer-Wilmes, Laila Najjari, Elmar Stickeler, Julia Wittenborn

**Affiliations:** 1 Department of Gynecology and Obstetrics, University Hospital RWTH Aachen, 52074 Aachen, Germany; rsenger@ukaachen.de (R.S.); phmeyer@ukaachen.de (P.M.-W.); lnajjari@ukaachen.de (L.N.); estickeler@ukaachen.de (E.S.); juwittenborn@ukaachen.de (J.W.); 2 Department of Economics and Business Administration, University of Applied Sciences Stralsund, Zur Schwedenschanze 15, 18435 Stralsund, Germany; lieven.kennes@hochschule-stralsund.de

**Keywords:** endometriosis, EHP-30, quality of life, lower abdominal pain, influencing factors

## Abstract

**Background/Objectives**: Endometriosis is a chronic gynecological disorder characterized by ectopic endometrial-like tissue. The symptoms of this disease negatively affect the patient’s quality of life, both physically and mentally. This study aims to identify key factors impacting health-related quality of life in endometriosis patients. **Methods**: A total of 175 patients from the Endometriosis Centre of the RWTH Aachen University Hospital were assessed using the German version of the EHP-30. The EHP is a patient self-report tool used to measure the wide range of impacts that endometriosis can have on women’s lives (affecting pain levels, leading to feelings of powerlessness and a loss of control, and affecting their emotional well-being, social support, and self-image). Multivariate linear regression and random forest analyses were performed to evaluate predictors of health-related quality of life, focusing on demographic characteristics, pain severity, endometriosis symptoms and planned procedures. **Results**: Key factors that have a significant negative impact on QoL include higher pain scores, dysuria, and persistent endometriosis. Higher pain scores negatively affect the EHP-30 pain (*p* < 0.0001), control and powerlessness (*p* < 0.0001) and emotional well-being (*p* < 0.01) scores. Dysuria has a negative effect on pain (*p* < 0.001), control and powerlessness (*p* < 0.001), emotional well-being (*p* < 0.05), and social support (*p* < 0.05). Persistent endometriosis was negatively associated with pain (*p* < 0.01), control and powerlessness (*p* < 0.01), and social support. Previous endometriosis surgery has a positive effect on the EHP-30 scores for pain, control and powerlessness, emotional well-being, and self-image (*p* < 0.05). **Conclusions**: Our study highlights the multifactorial impact of endometriosis on health-related QoL. Personalized treatments focusing on pain management, emotional support and social interventions are crucial to improve patient outcomes.

## 1. Introduction

Endometriosis is a complex, chronic gynecological disease characterized by the presence of endometrial-like tissue outside the uterus and is associated with symptoms which significantly reduce the quality of life (QoL) of affected women [1,2].

Endometriosis-related pain often limits work, social, and family activities [2]. The impact of endometriosis on quality of life is one of the key issues associated with the condition, as the impact of the disease goes far beyond its physical symptoms. It has been shown that women with endometriosis often suffer from psychological distress, including depression, anxiety, and stress, which can severely limit their daily lives and social interactions [3,4,5]. These psychological burdens are not only direct consequences of the chronic pain and other physical symptoms but are also exacerbated by the social stigmatization and lack of understanding of the condition. Delayed recognition and late diagnosis of endometriosis, which on average takes up to 10 years, are thought to play central roles in exacerbating these negative psychological and physical effects [6].

It is estimated that approximately 10–15% of women of reproductive age worldwide suffer from endometriosis, highlighting the significant health and socioeconomic burden of this disease [7,8].

Research into quality of life in endometriosis patients has become a focus of attention in recent years. It has been recognized that conventional medical and surgical treatments alone are often insufficient to address the overall impact of the disease. Loss of income due to frequent sick leave, high healthcare costs associated with treatments, and difficulty affording specialized care can significantly impact the QoL of women with endometriosis [9]. In addition, psychosocial factors such as partner and family support and access to specialized endometriosis practices or clinics play crucial roles in helping patients to cope with the disease and maintain an acceptable quality of life [10].

The aim of this study is to identify the most important factors affecting quality of life in women with endometriosis. The impacts of a wide range of endometriosis symptoms, previous endometriosis treatments, and different treatment plans on QoL are investigated. We present the assessment of quality of life using the well-validated Endometriosis Health Profile Questionnaire in long-form 30 (EHP-30) item instrument in patients attending our endometriosis centre for diagnosis and treatment of this disease. We use multivariate linear regression and machine learning methods to assess the most important factors influencing health-related quality of life in patients with endometriosis.

## 2. Materials and Methods

### 2.1. Study Design and Population

Data from a total of 175 patients who were outpatients with lower abdominal pain or suspicion of endometriosis at the endometriosis centre of RWTH Aachen University Hospital between October 2022 and December 2023 were analyzed.

Prior to the examination, patients completed a standardized medical history form in the waiting room, including the German version of the EHP-30 [11]. The EHP-30 is a well-evaluated questionnaire that is used in research and clinical settings to assess the quality of life of endometriosis patients. This core self-report instrument has five scale scores covering pain (11), control and powerlessness (6), social support (4), emotional well-being (6) and self-image (3). The numbers in brackets indicate the number of items in each scale of the core EHP-30. Response categories are rated on a five-point Likert scale (0–4). The purpose of the EHP-30 is to indicate the level of self-reported ill health in each domain measured. Therefore, each scale is standardized on a scale of 0–100, with 0 representing the best health and 100 the worst health. Scale scores for each scale are calculated as the sum of the raw scores of each item in the scale divided by the maximum possible raw score of all items in the scale multiplied by 100. Published evidence concludes that the EHP-30 is a robust and relevant tool for assessing areas of concern for women with endometriosis that are not covered by other condition-specific and generic questionnaires. The EHP-30 is the most reliable and thoroughly validated questionnaire for measuring health-related quality of life (HRQoL) in women with endometriosis [11,12,13]. It has good reliability, validity, and interpretability [11,13,14,15] and has been recommended for use in endometriosis HRQoL research by both the American Society for Reproductive Medicine and the European Society for Human Reproduction and Embryology [16].

The diagnosis of endometriosis was made according to the current ESHRE guidelines [17] after a gynecological examination and ultrasound; these were performed by a senior consultant with extensive experience at a specialized endometriosis centre. To assess the clinical diagnosis and location of endometriosis, we used the #ENZIAN scoring system [18]. The examination was followed by a detailed medical consultation, during which the doctor and the patient discussed further treatment planning, as well as a counselling and support session on the topic of endometriosis.

A standardized medical history form and a medical interview were used to record demographic data and self-reported symptoms. A medical examination report was used to record clinical data in detail. Pain severity, typical endometriosis symptoms, main complaints, operations, endometriosis diagnosis and planned procedures were assessed in all patients.

Pain severity was assessed using the visual analogue scale (VAS), which is a well-validated rating scale that is commonly used in epidemiological and clinical research to measure the intensity or frequency of different symptoms [19]. We used the VAS on a 10-point scale (0—no pain; 10—extreme pain) to assess the intensity of pain due to endometriosis.

The presence of typical endometriosis symptoms such as dysmenorrhoea, dysuria, dyschezia, and dyspareunia was clarified. The main reason for the patient’s presentation at the endometriosis centre was determined. A distinction was made between pain, persistent endometriosis, recurrent endometriosis, unfulfilled desire to have children, findings requiring clarification, and follow-up.

After the examination, endometriosis was diagnosed as peritoneal endometriosis, ovarian endometriosis, deep infiltrating endometriosis, or adenomyosis uteri. Following the presentation at the endometriosis centre, concrete endometriosis-specific therapy was planned; this included endocrine therapy, surgical therapy, analgesia, or reproductive therapy.

### 2.2. Statistical Analysis

Continuous variables are expressed as mean values ± standard deviation (SD). Categorical data are presented by absolute frequencies and percentages. To identify the most meaningful predictor variables for QoL in women with endometriosis, multivariate linear regression as well as random forests from the AI-toolbox were performed.

For multivariate linear regression, a full model that initially included all variables was utilized to investigate their influence on the different EHP-scale scores. Model selection was performed using the Akaike information criterion (AIC) [20]. The AIC is defined by 2k−2ln(L), where k is the number of predictor variables and L is the maximum value of the likelihood function of the linear regression model. The better the model fits the data, the higher the value of the likelihood function and, thus, the lower the AIC. Models with lower AIC are better. The number of predictor variables affects the AIC positively; thus, the 2k-term is often referred to as a “penalty term”, penalizing the addition of extra variables to discourage overfitting. The best-fitting model, with the lowest AIC, is reported in the Results section. Multicollinearity was investigated by variance inflation factors (VIFs). In the final parsimonious models, all the VIFs are significantly below 5, and in most cases, they are below 2.

A second method, supervised machine learning—more specifically random forest—was used for the identification of meaningful predictor variables and for predicting EHP-scale scores. We used random forest with variable importance in the field of explainable artificial intelligence (XAI) for several reasons. The advantage of random forest compared to multivariate linear regression is that random forest also recognizes non-linear relationships. Moreover, in the field of machine learning/artificial intelligence, random forest with variable importance yields higher explainability, an important feature in AI, than many black box algorithms, like, e.g., neural networks.

To assess the performance of prediction for unseen data for both methods, the ‘Leave-one-out’ cross-validation-procedure was used. For each subject, the model (regression or random forest) was derived based on the remaining 174 subjects and the resulting model utilized to predict the EHP-scale score for the subject, which was left out during the training. This procedure was repeated for all 175 subjects. The errors between these 175 predictions and the real EHP-scale scores were determined for all 175 subjects and averaged (mean absolute error—MAE). Thus, the MAE can be interpreted as the expected error (deviation on the 100-point scale) the model makes when predicting EHP-QoL-scores of new patients. Additionally, the root mean squared error (RMSE) was determined as the second performance metric.

*p* < 0.05 was considered statistically significant. Because of the exploratory nature of the study, the significance level was not adjusted for multiplicity. The statistical analyses were conducted using the statistical software R version 4.3.0 [21].

## 3. Results

A total of 175 patients who were diagnosed with endometriosis at the Endometriosis Centre of the University Hospital RWTH Aachen were included in this study (Table 1). The mean age of the patients was 27.8 years (SD 8.79). Most of the women (60%, *n* = 105) had already been diagnosed with endometriosis before they presented to our department. The main symptom of endometriosis was dysmenorrhea (96.6%, *n* = 169), followed by dyspareunia (38.3%, *n* = 67). The majority of woman (95.4%, *n* = 167) complained of pain. Of the 105 patients who had previously been treated for endometriosis, *n* = 60 (34.3% of the total patient population) had undergone surgery. The main suspected localizations of endometriosis, based on clinical findings, were peritoneum (63.4%, *n* = 111) and adenomyosis uteri (42.3%, *n* = 74). After careful examination in our department, the recommended therapy was, in most cases, endocrine therapy (72.6%, *n* = 127). Surgery was recommended in only (35.4%, *n* = 62) of patients.

**Figure 1:**
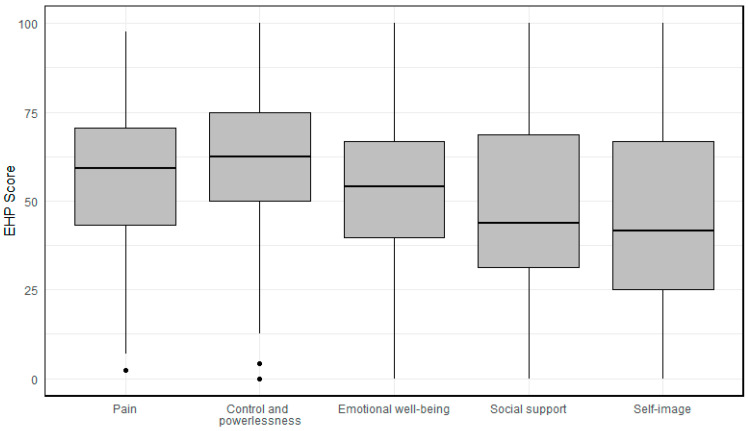
Mean and standard deviation on the Endometriosis Health Profile-30 core scales.

The mean ± standard deviation (SD) for the core scale scores of the EHP-30 was as follows: pain, 56.3 ± 20.9; control and powerlessness, 60.8 ± 21.9; emotional well-being, 52.5 ± 19.7; social support, 46.4 ± 25.2; and self-image, 46.4 ± 26.2 (Figure 1).

The results of the multivariate analysis using linear regression are shown in Table 2. To avoid over-parameterization of the model, a parsimonious model was determined in the multivariate linear regression. We used the parsimonious model separately for each scale score of the EHP-30 (pain, control and powerlessness, social support, emotional well-being, and self-image). The variables medical history, typical symptoms of endometriosis, main complaints, previous surgery, location of endometriosis, and planned therapy were included in each model.

The models pain (MAE: 13.68), control and powerlessness (MAE: 15.65), and emotional well-being (MAE: 15.07) are robust and show good performance. The performances of the emotional well-being (MAE: 19.42) and self-image (MAE: 22.39) models are poorer.

Random forest showed overall lower model quality values than multivariate linear regression for each EHP-30 score (pain, control and powerlessness, emotional well-being, social support and self-image) (Table 3).

### 3.1. Pain

For the EHP-30 pain score, the best-fitting model included the variables visual analogue scale pain (VAS), dysuria, pain as the main complaint, persistent endometriosis, desire to have children, and previous endometriosis surgery. The variables included in the model that have a significant impact on a higher pain score on the EHP-30 are VAS (*p* < 0.0001), dysuria (*p* < 0.0001), and persistent endometriosis (*p* < 0.01). Patients with a desire to have children (*p* < 0.001) have significantly lower EHP-30 pain scores.

### 3.2. Control and Powerlessness

Regarding the influence on the EHP-30 control and powerlessness scores of the EHP-30, the best-fitting model included the variables VAS, dysmenorrhoea, dysuria, follow-up, persistent endometriosis, previous abdominal surgery, previous endometriosis surgery, peritoneal endometriosis and adenomyosis uteri. The variables included in the model that have a significant negative impact on the control and powerlessness aspect of the EHP-30 are VAS (*p* < 0.0001), dysuria (*p* < 0.001), follow-up (*p* < 0.05), persistent endometriosis (*p* < 0.01), and peritoneal endometriosis (*p* < 0.05). The variables dysmenorrhoea (*p* = 0.151) and previous endometriosis surgery (*p* = 0.152) included in the model have a positive but not statistically significant effect on the EHP-30 control and powerlessness scores.

### 3.3. Emotional Well-Being

For the EHP-30 emotional well-being score, the best-fitting model included the variables age, VAS, dysuria, pain as the main complaint, findings requiring clarification, recurrence, previous abdominal surgery, previous endometriosis surgery, ovarian endometriosis, and reproductive medicine as the suggested procedure. Analysis of the variables included in the emotional well-being model revealed significantly higher scores for VAS (*p* < 0.01), dysuria (*p* < 0.05), previous abdominal surgery (*p* ≤ 0.01), and ovarian endometriosis (*p* < 0.05).

Significantly lower EHP-30 emotional well-being scores were found for the variable age (*p* < 0.05).

### 3.4. Social Support

The best-fitting model included the variables dysuria, persistent endometriosis, recurrence, ovarian endometriosis, surgical therapy, and endocrine therapy as suggested procedures for the EHP-30 social support score. Significant negative effects of the EHP-30 social support score were found for the variables dysuria (*p* < 0.05), ovarian endometriosis (*p* ≤ 0.05), and surgical therapy (*p* < 0.001). Only the variable recurrence (*p* < 0.05) has a significant positive effect on EHP-30 social support.

### 3.5. Self-Image

For the EHP-30 self-image score, the best-fitting model included first diagnosis, previous abdominal surgery, and previous endometriosis surgery. The higher EHP-30 self-image score was significantly affected by previous abdominal surgery (*p* ≤ 0.01) and significantly positively influenced by previous endometriosis surgery (*p* < 0.05).

### 3.6. Summary

Some variables generated in the parsimonious model for each core of the EHP-30 are repetitive and have the same influence on the score. The visual analogue score for pain is represented in the pain, control and powerlessness, and emotional well-being models and has a significant negative effect on the score. Similar results are shown for the variable dysuria, which has a significant negative effect on pain, control and powerlessness, emotional well-being, and social support. Persistent endometriosis has a negative impact on the EHP-30 scores for pain, control and powerlessness, and social support.

In contrast, previous endometriosis surgery was presented as a variable with a positive effect on the EHP-30 scores for pain, control and powerlessness, emotional well-being, and self-image scores. However, the effect was only significant for self-image.

As noted earlier, the performance of the random forest model was slightly inferior across all five EHP-30 scores for both performance metrics (Table 3). However, the comparable magnitude of error metrics, along with the insights from variable importance analysis, underscores the robustness of the results obtained by multivariate linear regression.

## 4. Discussion

The aim of our study was to investigate the relationship between endometriosis and quality of life and to examine the main factors influencing health-related quality of life in patients attending our endometriosis centre. We found several correlating factors focusing on different aspects of quality of life, including pain, control and powerlessness, social support, emotional well-being, and self-image.

This study provides valuable insights into the complex factors affecting the quality of life in women with endometriosis. By utilizing the Endometriosis Health Profile Questionnaire (EHP-30), this study underlines how pain, emotional distress, and social support deficits significantly impact the lives of women with endometriosis.

We were able to show that pain is the key influencing factor concerning endometriosis patients’ health-related quality of life. Higher visual analogue scale (VAS) pain scores are strongly associated with worse quality-of-life outcomes across multiple domains, particularly pain, control and powerlessness, and emotional well-being. The significant role of pain in quality-of-life outcomes emphasize how endometriosis with severe pain leads to severe physical and psychological consequences. These findings highlight the need to target psychological factors in the treatment of women with endometriosis [22]. Similarly, Vercellini et al. [1] showed that pain plays a central role in endometriosis. In total, 95.3% (*n* = 167) of the patients included in the study suffer from pain. This supports research by Facchin et al. [3], who highlighted that chronic pain from endometriosis often results in psychological burdens such as anxiety and depression. The relationship between pain and emotional well-being supports the need for comprehensive pain management as part of endometriosis treatment and should be put at the centre of treatment strategies.

We found that persistent endometriosis worsens pain, control and powerlessness, and social support. This suggests that inadequate prior treatment of the disease plays a central role in reducing quality of life. Patients with persistent endometriosis (*n* = 25) presenting to our clinic had been treated for endometriosis in the past, which included undergoing surgical removal of the endometriosis (*n* = 24, 96%). In most cases, previous treatment had been unsuccessful. Unfortunately, only about 40% of patients with endometriosis are treated in specialized facilities such as endometriosis centres or clinics [23]. As a result, patients are often not treated according to current guidelines, and surgical treatment of endometriosis is incomplete, often leading to a high rate of unnecessary re-operation in 27–58% [24,25,26]. This lack of optimal treatment as the main reason for persistent endometriosis often leads to frustration and chronic pain for patients with endometriosis and has a negative impact on their quality of life.

Another important finding of this study is the significant negative impact of dysuria, which affects multiple domains such as pain, emotional well-being, and social support. Endometriosis that spreads and invades the urinary tract is rare, occurring in 0.3–12% of all endometriosis patients [27,28]. However, the symptom of dysuria is more common. Although this symptom is common, occurring in 15–50% of patients with endometriosis, dysuria is a less-studied symptom in endometriosis research. It has previously been noted that dysuria appears to exacerbate the overall disease burden [29,30], which is in line with our findings.

Ovarian endometriosis is not only associated with the typical symptoms of endometriosis, but also with the loss of follicles. Infertility or subfertility and concerns about potential infertility are a major source of worry or depression [31,32]. This is particularly important, as there is evidence that the extent of AMH (anti-Müllerian hormone) decline after endometrioma surgery varies significantly depending on the characteristics of the endometrioma, surgical techniques, and hemostatic methods [33,34,35]. In our view, fertility-related anxiety explains why patients with ovarian endometriosis have higher EHP-30 scores for emotional well-being and social support.

Previous surgery for endometriosis was included as a variable in four of the five best-fitting models of the EHP-30. The positive effect of this variable was only significant for self-image but tended to have a positive effect on scores for pain, control and powerlessness, and emotional well-being. Drechsel-Grau et al. [36] also show the positive effect of surgery in patients with deep infiltrating endometriosis in reducing pain and improving fertility in most cases. In addition to endocrine therapy, surgical therapy is one of the basic treatment options for endometriosis. We assume that the reason for this finding in our study is that correctly indicated and performed surgery for endometriosis reduces the symptoms of endometriosis and improves the quality-of-life domains, as has been shown previously [37].

This study also highlights the positive impact of the desire to have children on the EHP-30 pain scores. Women who wished to have children reported lower pain scores, which may reflect a psychological focus on reproductive outcomes over physical pain. Endometriosis can lead to infertility, including reduced likelihood of conception after in vitro fertilization [38,39]. The patients who presented at our endometriosis centre because of infertility did not necessarily have symptoms of endometriosis in the sense of pain (dysmenorrhoea, dyspareunia, dysuria or dyschezia), meaning that the pain component plays a secondary role.

In addition, our study highlights the role of social support, which is crucial for managing the psychological burden of endometriosis. The social isolation that can occur due to the stigma of endometriosis, combined with the chronic nature of the disease, exacerbates feelings that have a critical negative impact on quality of life [2]. As shown in our results, dysuria, ovarian endometriosis and need for surgical interventions were negatively associated with social support, while recurrence was positively linked to better social support, possibly indicating greater involvement of support networks, such as family or a partner, in recurrent cases.

In our analysis, linear regression outperformed the random forest model potentially due to a predominantly linear relationship between the independent variables and the target outcome, making the simpler linear model a more suitable choice. Additionally, given the limited sample size, linear regression was less prone to overfitting and generalized better, particularly in the presence of noisy data where random forests tended to capture irrelevant fluctuations.

The strength of this study is that we used a validated instrument to assess quality of life in a large sample of women with endometriosis, which was analyzed using a robust analytical approach to identify factors affecting quality of life. These findings may improve the management of endometriosis through more personalized treatment planning.

The limitation of assessing quality of life in endometriosis patients was that we used a single quality-of-life instrument in our study. As highlighted in the review by Jones GL et al. [13], although the EHP-30 is reliable and responsive, its lengthy nature and limited cross-cultural validation in diverse populations may limit its applicability in some settings.

The limitation of this study is that the complex psychological, social, and economic factors are not fully captured by the variables included in this study, as they are not commonly part of the presentation of the patient in the endometriosis centre. This could explain the lower performance of the models in relation to emotional well-being and self-image. Future research should consider integrating measures of psychological resilience, financial burden, and access to specialized care for endometriosis patients.

## 5. Conclusions

In conclusion, this study highlights the multifactorial nature of endometriosis and its significant impact on women’s quality of life. Pain, emotional distress, and lack of social support are interrelated, and comprehensive management strategies must address both the physical and psychological aspects of the disease. In addition to medical and surgical treatments, psychological support, social interventions and improved diagnostic pathways are essential to improve the overall well-being of women with endometriosis. This supports the need for multimodal therapy of endometriosis. Future research should focus on integrating a broader range of psychosocial factors, including financial burden and access to care, to better understand the full impact of endometriosis on quality of life.

The clinical impact of this study is that we have identified the most important factors that negatively and positively affect quality of life in women with endometriosis, such as pain severity (measured by VAS), dysuria, and persistent endometriosis. By identifying these variables, clinicians can prioritize patient concerns and symptoms that most affect daily functioning and overall well-being. This understanding can lead to more targeted, personalized treatments.

## Figures and Tables

**Table 1:** Baseline characteristics.

	Mean (SD)
Age	27.8 (8.79)
Pain severity (Visual analogue scale)	7.25 (1.87)
	**Number of patients *n* (%)**
First diagnosis	70 (40.0)
Previous therapy	105 (60.0)
Current therapy	
None	99 (56.6)
Endocrine therapy	63 (36.0)
Analgesia	13 (7.4)
**Typical endometriosis symptoms**	
Dysmenorrhoea	169 (96.6)
Dyspareunia	67 (38.3)
Dysuria	18 (10.3)
Dyschezia	33 (18.9)
**Main complaints**	
Pain	167 (95.3)
Findings requiring clarification	4 (2.3)
Follow-up	9 (5.1)
Persistent endometriosis	25 (14.3)
Recurrence	23 (13.1)
Desire to have children	34 (19.4)
**Operations**	
Previous abdominal surgery	78 (44.6)
Previous endometriosis surgery	60 (34.3)
Previous histological confirmation of endometriosis	58 (33.1)
**Location of endometriosis diagnosis**	
Peritoneal (#ENZIAN P)	111 (63.4)
Ovary (#ENZIAN O)	10 (5.7)
Deep infiltrating endometriosis (#ENZIAN A/B/C)	20 (11.4)
Adenomyosis uteri (#ENZIAN FA)	74 (42.3)
**Planned procedure**	
Surgical therapy	62 (35.4)
Drug-based pain therapy/analgesia	17 (9.7)
Reproductive medicine	5 (2.9)
Endocrine therapy	127 (72.6)

**Table 2:** Results of multivariate linear regression with variables included in the best-fitting model of the EHP-30 for each core instrument.

			Multiple R^2^
Pain	Estimates	*p*-Values	R^2^ = 0.40
**Visual analogue scale pain**	6.27	<0.0001	
**Dysuria**	15.10	<0.001	
Pain	−11.55	0.133	
**Persistent endometriosis**	11.69	<0.01	
**Desire to have children**	−11.13	<0.001	
Previous endometritis surgery	−5.56	0.087	
**Control and powerlessness**			R^2^ = 0.32
**Visual analogue scale pain**	5.63	<0.0001	
Dysmenorrhoea	−13.47	0.151	
**Dysuria**	16.07	<0.001	
**Follow-up**	17.51	<0.05	
**Persistent endometriosis**	15.40	<0.01	
Previous abdominal surgery	8.63	0.079	
Previous endometriosis surgery	−8.03	0.152	
**Peritoneal endometriosis**	7.91	<0.05	
Adenomyosis uteri	5.24	<0.01	
**Emotional well-being**			R^2^ = 0.18
**Age**	−0.38	<0.05	
**Visual analogue scale pain**	3.00	<0.01	
**Dysuria**	10.48	<0.05	
Pain	−14.60	0.095	
Findings requiring clarification	14.28	0.147	
Recurrence	−7.41	0.150	
**Previous abdominal surgery**	14.16	<0.01	
Previous endometriosis surgery	−9.43	0.084	
**Ovary**	16.09	<0.05	
Reproductive medicine	12.62	0.141	
**Social support**			R^2^ = 0.14
**Dysuria**	14.57	<0.05	
Persistent endometriosis	8.04	0.129	
**Recurrence**	−14.21	<0.05	
**Ovary**	19.03	<0.05	
**Surgical therapy**	15.92	<0.001	
Endocrine therapy	9.84	0.053	
**Self-image**			R^2^ = 0.052
First diagnosis	−6.89	0.156	
**Previous abdominal surgery**	18.90	<0.01	
**Previous endometriosis surgery**	−18.89	<0.05	

**Table 3:** Comparison of model performance (multivariate linear regression vs. random forest) using MAE and RMSE.

	MAE (Mean Absolute Error)/Leave One Out		RMSE (Root Mean Square Error)/Leave One Out	
	Performance Metrics Multivariate Linear Regression	Performance Metrics Random Forest	Performance Metrics Multivariate Linear Regression	Performance Metrics Random Forest
Pain	13.68	14.25	16.80	17.70
Control and Powerlessness	15.65	16.55	19.54	20.51
Emotional well-being	15.07	15.79	19.04	19.90
Social support	19.42	19.77	24.28	24.75
Self-image	22.39	22.40	26.05	26.06

## Data Availability

The datasets generated during the current study are available from the corresponding author on reasonable request.
